# Cannabis-involved Substance Use Treatment Admissions Age 50+, 2000-2021: Changes and Correlates

**DOI:** 10.1093/geroni/igaf122.2258

**Published:** 2025-12-31

**Authors:** Namkee Choi, C Nathan Marti

**Affiliations:** The University of Texas at Austin, Austin, Texas, United States; The University of Texas at Austin, Austin, Texas, United States

## Abstract

Given the rapidly increasing medicinal and recreational cannabis use among U.S. older adults, we examined older adults’ cannabis-involved substance use treatment admissions. We used the 2000-2021 concatenated Treatment Episode Data Set-Admissions (TEDS-A) age 50 + (N = 5,593,004) and joinpoint regression models to examine changes in cannabis-involved admissions and multinomial and binary logistic regression models to examine the demographic and treatment-related correlates of cannabis-primary and -secondary/tertiary admissions. The results show that cannabis-involved admissions ages 50-64 increased significantly between 2000 and 2012 (annual percentage changes [APC]=4.36 and 6.62 for the 50-54 and 55-64 age groups, respectively) and then decreased between 2012-2021 (APC=1.84 and 1.27 for the 50-54 and 55-64 age groups, respectively). In the 65+ age group, there was a steady increase between 2000 and 2016 (APC=5.2), followed by no significant changes between 2016 and 2021. Compared to no-cannabis admissions, the likelihood of both cannabis-primary and -secondary/tertiary admissions was higher among males, Blacks, American Indians/Alaska Natives, residents of states where medical or recreational cannabis use was legal, and referrals from healthcare providers and court/criminal legal systems. The likelihood of cannabis-primary admissions was higher among those age 65+, Blacks, Hispanics, residents of states with medical cannabis laws, and those who were referred by healthcare providers and court/criminal legal systems. More effective regulations and enforcement of delta-9-tetrahydrocannabinol potency and research on cannabis harms and poly-substance use are needed to protect the health of older adults who turn to cannabis for its purported health benefits. Increased availability and accessibility of treatment infrastructure are also needed.

